# In This Issue

**Published:** 1994

## ALCOHOLISM TREATMENT IN THE UNITED STATES

Each year, approximately 600,000 clients are treated for alcoholism. Effective treatment results in decreased alcohol consumption and improved psychosocial functioning for these clients. Treatment also has been shown to reduce long-term medical costs for alcoholics by almost 25 percent. Drs. Mary E. McCaul and Janice Furst review the treatment options currently available in the United States. Various components of the traditional 12-step and inpatient and outpatient modalities are evaluated. These components range from brief interventions that can be provided by a physician during an office consultation to medications that reduce the craving for alcohol, such as the drug naltrexone. Not all treatment modalities work equally well for all clients. Research is underway to determine how treatment can be tailored specifically to meet the needs of the individual. (pp. 253–260)

## PSYCHIATRIC COMORBIDITY

The occurrence of psychiatric disorders and alcoholism in the same patient presents problems for clinicians and patients alike. Co-occurring (comorbid) disorders are poorly understood and frequently misdiagnosed, and their treatment is problematic. Alcoholics with psychiatric disorders may fall through the cracks in the health care system, failing to receive treatment for one or the other of their disorders. Fortunately, prospects for effective treatment are improving with the increasing integration of psychiatric and addiction treatment perspectives. Dr. Norman S. Miller discusses some problems in recognizing addictive-psychiatric comorbidity and evaluates current treatment models. (pp. 261–264)

## MEDICATIONS FOR TREATING ALCOHOLISM

Researchers continue to evaluate medications to assist in the treatment of alcoholism. Dr. Raymond F. Anton provides an overview of the medications intended to reduce the craving for alcohol, treat psychiatric disorders that may coexist with alcoholism, discourage alcohol consumption, antagonize alcohol’s effects, and ameliorate withdrawal symptoms. Medications that prove useful in treating these or other aspects of alcoholism may serve as adjuncts to standard psychosocial methods of alcoholism treatment. (pp. 265–271)

## NALTREXONE FOR THE TREATMENT OF ALCOHOLISM

The search for new medications that will reduce the craving for alcohol and will be useful as adjuncts to alcoholism therapy has resulted in clinical trials using naltrexone, a drug already prescribed in the treatment of opiate addiction. Dr. Joseph R. Volpicelli and his colleagues review early research of naltrexone that suggests how this substance might act in the brain. They also describe two human clinical trials using naltrexone that support its effectiveness in deterring relapse among recovering alcoholics. (pp. 272–278)

## COGNITIVE-BEHAVIORAL APPROACHES TO TREATMENT

In the cognitive-behavioral approach to alcoholism, pathological drinking is considered a learned behavior that is developed by a person to cope with problems or to fulfill certain needs. Dr. Ronald M. Kadden discusses approaches to alcoholism treatment that are based on this view. He describes how clients can be taught to modify their behaviors and build a social support network to help them achieve and maintain abstinence. (pp. 279–286)

## PATIENT-TREATMENT MATCHING

Because no single treatment is effective for all individuals with alcohol problems, researchers now are focusing on matching each patient with the most appropriate treatment. Dr. Margaret E. Mattson defines patient-treatment matching and highlights the search for guidelines that will predict the most effective treatments for many types of patients. She explains how treatment providers might use these guidelines as blueprints for determining the best treatment for each client. Dr. Mattson also outlines the National Institute on Alcohol Abuse and Alcoholism’s ongoing treatment-matching research project, Project MATCH. (pp. 287–295)

## TREATMENT OF ADOLESCENT ALCOHOL ABUSE AND DEPENDENCE

At least 6 percent of the clients in alcoholism and drug abuse treatment programs today are under 18 years of age. Effective diagnosis of adolescents with alcohol problems is important. However, Dr. Oscar G. Bukstein contends that because most diagnostic criteria have been established for use with adults, they may not be applicable to adolescents. He also examines the special needs of adolescents in alcoholism treatment and suggests how their care can be improved. For example, the treatment design should take into consideration that the adolescent is a dependent family member undergoing physical and social maturation and susceptible to peer pressure. (pp. 296–301)

## MANDATED TREATMENT

The number of individuals with alcohol problems who are mandated to receive treatment has increased over the past three decades. Dr. Elisabeth Wells-Parker explores this trend by reviewing court-mandated treatment for drinking drivers in the United States. She describes the most common types of treatment for these offenders and reviews which types have been found most effective in reducing drinking and driving recidivism. She also notes the importance of offering incentives that encourage offenders to attend and complete treatment. Such incentives include levying fines or sanctioning the removal of drivers’ licenses when offenders fail to attend treatment programs. (pp. 302–306)

